# Effects of Tyrosine and Tryptophan Supplements on the Vital Indicators in Mice Differently Prone to Diet-Induced Obesity

**DOI:** 10.3390/ijms22115956

**Published:** 2021-05-31

**Authors:** Ivan V. Gmoshinski, Vladimir A. Shipelin, Nikita V. Trusov, Sergey A. Apryatin, Kristina V. Mzhelskaya, Antonina A. Shumakova, Andrey N. Timonin, Nikolay A. Riger, Dmitry B. Nikityuk

**Affiliations:** 1 Federal Research Centre of Nutrition and Biotechnology, 109240 Moscow, Russia; gmosh@ion.ru (I.V.G.); nikkitosu@yandex.ru (N.V.T.); apryatin@mail.ru (S.A.A.); kristik13@yandex.ru (K.V.M.); antonina_sh@list.ru (A.A.S.); andrey8407@mail.ru (A.N.T.); n_rieger@icloud.com (N.A.R.); dimitrynik@mail.ru (D.B.N.); 2 Academic Department of Innovational Materials and Technologies Chemistry, Plekhanov Russian University of Economics, 115093 Moscow, Russia; 3 Department of Operative Surgery and Topographic Anatomy, I.M. Sechenov First Moscow State Medical University (Sechenov University), 119991 Moscow, Russia

**Keywords:** obesity, mice, tyrosine, tryptophan, cytokines, behavior, cytokines, inflammation, liver morphology

## Abstract

We studied the effects of the addition of large neutral amino acids, such as tyrosine (Tyr) and tryptophan (Trp), in mice DBA/2J and tetrahybrid mice DBCB receiving a high-fat, high-carbohydrate diet (HFCD) for 65 days. The locomotor activity, anxiety, muscle tone, mass of internal organs, liver morphology, adipokines, cytokines, and biochemical indices of animals were assessed. The Tyr supplementation potentiated increased anxiety in EPM and contributed to a muscle tone increase, a decrease in the AST/ALT ratio, and an increase in protein anabolism in both mice strains. Tyr contributed to a decrease in liver fatty degeneration and ALT reduction only in DBCB that were sensitive to the development of obesity. The addition of Trp caused an increase in muscle tone and potentiated an increase in anxiety with age in animals of both genotypes. Trp had toxic effects on the livers of mice, which was manifested in increased fatty degeneration in DBCB, edema, and the appearance of micronuclei in DBA/2J. The main identified effects of Tyr on mice are considered in the light of its modulating effect on the dopamine neurotransmitter metabolism, while for the Trp supplement, effects were presumably associated with the synthesis of its toxic metabolites by representatives of the intestinal microflora.

## 1. Introduction

Genomic and post-genomic factors that determine the level of physical and metabolic activity play an important role in the development of obesity [1,2]. The development of methods for effective dietary correction of obesity in humans requires an understanding of the biological causes of decreased physical activity which lead to an imbalance between energy consumption and expenditure [3].

In this area, the study of the neuroendocrine regulation of metabolic processes [4] and the possible influence on them by pharmacological and dietary factors [5,6] is of particular interest.

The neurotransmitters dopamine [7], serotonin [8], and their minor metabolites (trace amines, etc.) [9] play a critical role in the regulation of energy metabolism, locomotor activity, and feeding behavior. Large neutral amino acids—tyrosine (Tyr) and tryptophan (Trp)—are precursors of these active metabolites. There is a definite link between changes in the signaling of dopamine in the striatum and motor activity [10]. In turn, the effect of serotonin on metabolic processes is due to the activation of the signaling pathway in the hypothalamus neurons that are part of the proopiomelanocortin system (POMC). Serotonergic neurons receive information on the composition of the diet through the amount of Trp entering the brain, which depends on the competition in transport across the blood-brain barrier between Trp, Tyr, and other large neutral amino acids (LNAA) (phenylalanine, leucine, isoleucine, methionine) [11].

The catabolism of tissue proteins containing a relatively small amount of Trp decreases under conditions of excessive carbohydrate and fatty nutrition. This leads in turn to an increase in the specific content of Trp in the whole pool of free amino acids of blood plasma and consequently an increase in its transport to the brain, where Trp is transformed into serotonin, which has anorexigenic and sedative effects [12].

In this regard, the question arises about the possibility of directed modulation of metabolic processes, physical activity, and eating behavior by changing the ratio between Trp and other LNAA (especially tyrosine) coming from food. This work aimed to study the effect of Tyr and Trp in the diet on the neuroendocrine regulation of metabolic processes in laboratory mice (*Mus domesticus*), using the assessment of behavioral reactions and integral, morphological, biochemical, and immunological parameters. Two strains of mice were used, of which the first, DBA/2J, is characterized by resistance to the development of diet-induced obesity. The second strain—a complex hybrid of the second generation obtained earlier in our laboratory—easily developed signs of diet-induced obesity and fatty liver degeneration when consuming a diet with excess fat and fructose, as was shown earlier in the study [13].

## 2. Results

### 2.1. Amino Acids Characterization

The possible presence of trace impurities in amino acids, especially in tryptophan preparation [14], that could influence their nutritive value and toxicity was checked by HPLC. As shown by the analysis, samples of tyrosine and tryptophan used in animal feeding did not contain any impurities detected within the method sensitivity (less than 0.1% by mass), which complies with the manufacturer’s specifications. 

### 2.2. Mice Integral Indices

**Figure 1:**
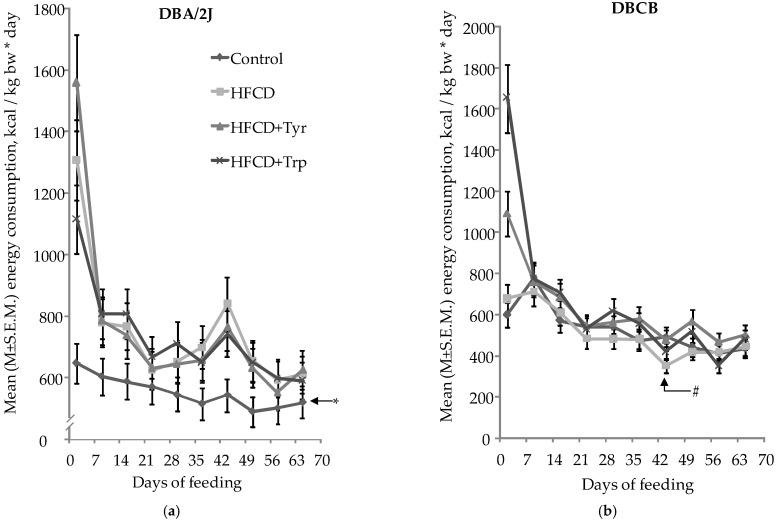
Specific energy consumption (M ± S.E.M.) of mice during the experiment: (**a**) DBA/2J mouse line; (**b**) DBCB mice. * The difference is significant with all groups receiving HFCD; # The difference is significant with group receiving HFCD with Tyr group, *p* < 0.05; Student’s *t*-test for pair-related, group-averaged indicators.

Assessment of integral indicators, such as specific energy consumption, body weight, internal organs, and tissues, shows the degree of diet-induced obesity in mice and allows assessment of the possibility of its alleviation by consuming the studied supplements. During almost the entire period of the experiment, DBA/2J mice of all groups stably increased their body weight (bw), had a normal appearance, and were mobile; morbidity and mortality were not observed. Among DBCB mice in group 2 fed with HFCD, 4 animals died during the experiment, with signs of necrotic myocardial changes; in groups 3 and 4, which received HFCD with Tyr and Trp, 1 animal died in each one. As follows from the data on Figure 1, DBA/2J mice of all groups receiving HFCD were characterized by higher energy consumption compared with the control group. In contrast, in tetrahybrid mice fed with HFCD, the total energy consumption did not increase compared to the control group since the mass of feed consumed by these animals was reduced in comparison with the control value, but the addition of tyrosine caused a significant increase in energy consumption in animals of group 3.

**Figure 2:**
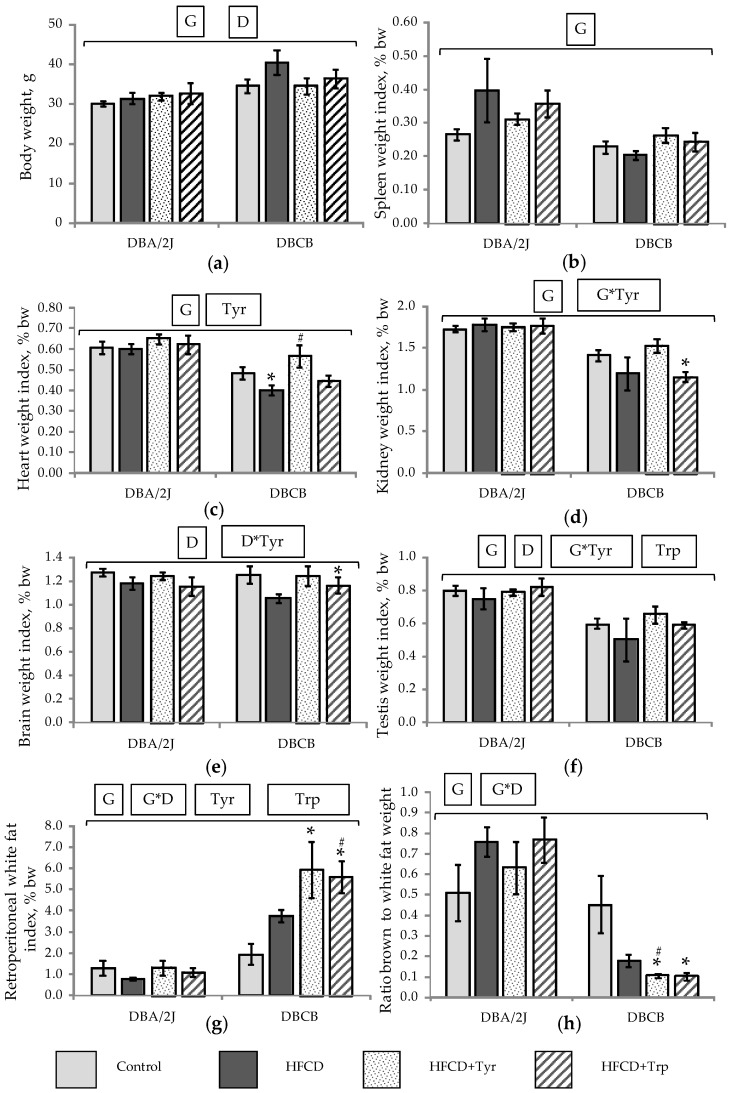
Integral indicators (M ± S.E.M.) of mice at the end of the experiment on the 66th day: (**a**) Bw; relative weight index of organs and tissues: (**b**) Spleen, (**c**) Heart, (**d**) Kidneys, (**e**) Brain, (**f**) Gonads, (**g**) Retroperitoneal white fat; (**h**) The ratio of the weight of interscapular brown fat to the retroperitoneal white fat. * The difference with the control group is significant; # The difference with the HFCD-only fed group is significant, *p* < 0.05, Mann–Whitney U-test. Horizontal bracket—distribution is non-uniform (*p* < 0.05, ANOVA) by the factors genotype (G), diet (D), tyrosine (Tyr), tryptophan, (Trp) and their combinations for the covered range of samples. The number of animals in groups—see Table 1.

At the end of the experiment, a significantly larger bw was observed in DBCB mice receiving HFCD compared with control, ANOVA, *p* < 0.05 by the diet (D) factor (Figure 2). The relative weight indexes of the spleen, heart, kidney, and gonads in the tetrahybrid mice were significantly lower, and retroperitoneal white fat was higher in comparison with mice DBA/2J, ANOVA, *p* < 0.05 by the genotype (G) factor. The addition of amino acids did not have a significant effect on bw, spleen, and gonad weight indexes, but in DBCB mice, Tyr consumption increased the relative weight of the heart, kidneys, and gonad to the level of the control group, and Trp caused a significant decrease in the weight of the kidneys and brain. Both amino acids in these mice had a potentiating effect on the accumulation of white fat and a decrease in the ratio of brown fat to white fat (see Figure 2h). In linear DBA/2J mice, there was no effect of amino acid additions on the weight indexes of organs and adipose tissue.

### 2.3. Muscle Tone, Search, and Anxiety-Like Behavioral Activity

**Figure 3:**
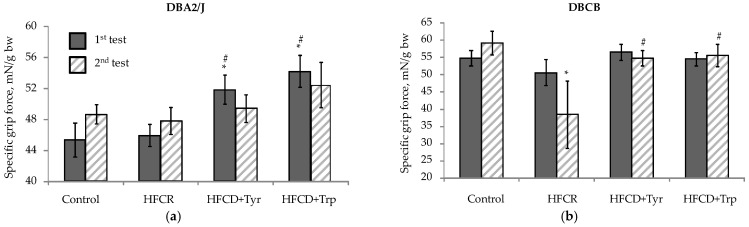
Muscle-contraction strength (grip force, M ± S.E.M.): (**a**) DBA/2J mice; (**b**) Tetrahybrid DBCB mice. * The difference with the control group is significant; # The difference with the HFCD-only group is significant, Mann–Whitney U-test.

Additional consumption of the amino acids tyrosine and tryptophan can lead to an increase in their transport to the brain, which may result in changes in the synthesis and metabolism of the neurotransmitter amines dopamine and serotonin as well as their derivatives. This is reflected by changes in the level of neuromotor activity, eating behavior, and anxiety. The definition of muscle-compressive force (Figure 3) showed that, in DBA/2J mice, even at the first test (the 3rd day of feeding with rations), the addition of both amino acids leads to an increase in muscle tone compared to the control and HFCD-fed group. Further, this effect is preserved only in the form of an insignificant trend. In DBCD tetrahybrid mice, when testing for the first time, the muscle-compressive force did not differ between the groups, while at the second test, it decreased significantly with the use of HFCD. Both amino acid supplements return this indicator to the control level.

**Figure 4:**
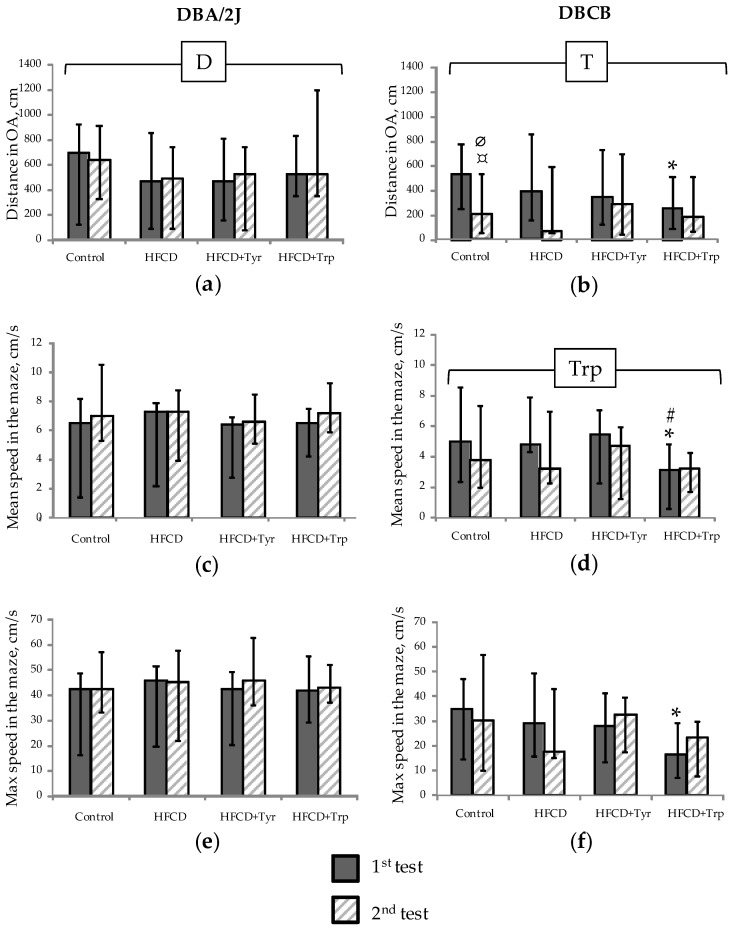
Mobility/locomotor activity indicators (median; change intervals) in the EPM test: (**a**,**b**) Distance traveled in the OA, cm; (**c**,**d**) Average speed in the maze, cm/s; (**e**,**f**) Maximum speed in the OA, cm/s. DBA/2J mice (**a**,**c**,**e**); Tetrahybrid DBCB mice (**b**,**d**,**f**). * The difference with the control group is significant, # the difference with the HFCD-only group is significant, ∅ the difference with DBA/2J mice is significant, *p* < 0.05, Mann–Whitney U-test; ¤ difference between 1st and 2nd test is significant, *p* < 0.05, Wilcoxon test for pairwise related values. Horizontal bracket—distribution is non-uniform (*p* < 0.05, ANOVA) by factors diet (D), test (T), and tryptophan (Trp) for the covered sample range. The number of animals in groups—see Table 1.

Study of locomotor/search activity in elevated plus maze test (EPM) (Figure 4) showed no effect of HFCD and amino acid additives on these indicators in DBA/2J mice (Figure 4a,c,e) except for the general decrease in the distance traveled in open arms (OA) (Figure 4a) for all animals that received HFCD. In DBCB mice, locomotor activity in the OA decreased significantly with repeated testing (Figure 4b), with the most pronounced change in the control group. The addition of Trp in these mice led to a significant decrease in the average movement speed (Figure 4d) and maximum speed in the OA (Figure 4f) during the first testing. When comparing two animal lines, attention is generally paid to the lower mobility of tetrahybrid mice compared to DBA/2J, which is confirmed by the data of factor analysis (*p* < 0.05 by the genotype factor for speed in the OA at 2nd test).

**Figure 5:**
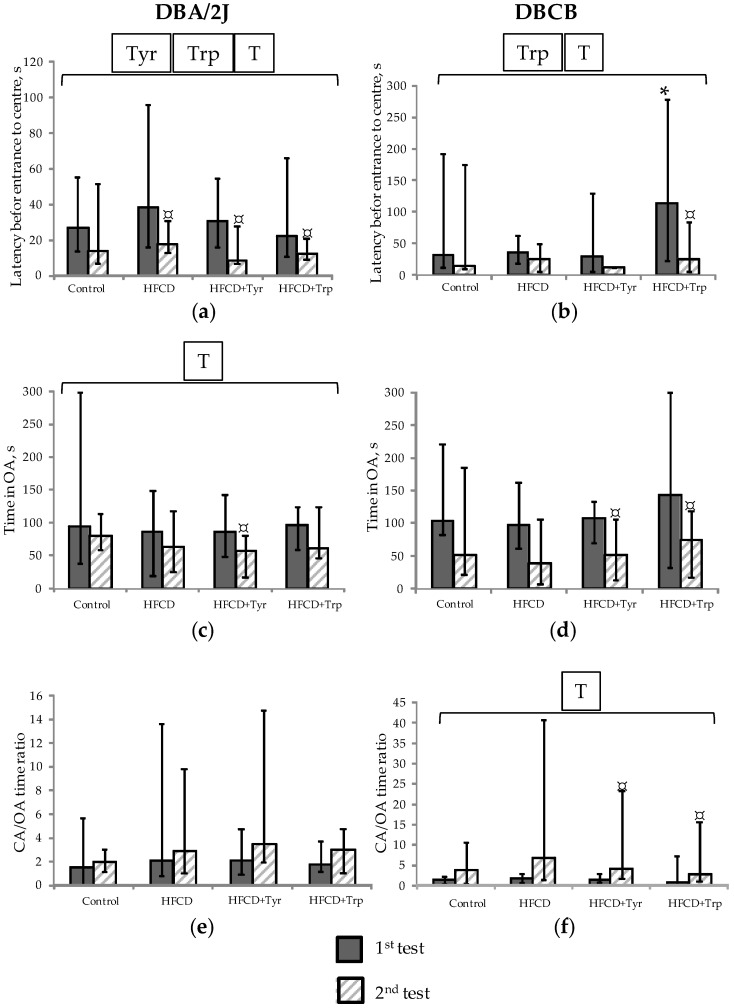
Indicators of anxiety (median; change intervals) of mice in the EPM test: (**a**,**b**) Latency before the first exit to the center of the maze, s; (**c**,**d**) Time in OA, s; (**e**,**f**) The ratio of CA/OA times (dimensionless). DBA/2J mice (**a**,**c**,**e**); Mice DBCB tetrahybrid (**b**,**d**,**f**). * The difference with the control group is significant; # The difference with the group fed HFCD only is significant, *p* < 0.05, Mann–Whitney U-test; ¤ The difference between the 1st and the 2nd tests is significant, *p* < 0.05, Wilcoxon test for pairwise related values. Horizontal bracket—distribution is non-uniform (*p* < 0.05, ANOVA) by the factors test (T), tyrosine (Tyr), and tryptophan (Trp) for the covered range of samples. The number of animals in groups—see Table 1.

The anxiety-like behavior in mice, detected in EPM (Figure 5), increased in the 2nd testing according to parameters of the center entrance latency, time spent in OA, and the ratio of closed arms (CA) to OA times. These findings coincide with the previously obtained data from C57Black/6J mice, which represent a parental line for DBCB tetrahybrid [15]. Anxiety increase most clearly manifested in a decrease in the latency time to the first exit to the maze center, the time spent in the OA in DBA/2J mice, and an increase in the CA/OA ratio in DBCB tetrahybrid (*p* < 0.05, ANOVA, by the test factor). At the same time, the addition of Tyr to the diet caused a significant decrease in the OA time in mice of both strains during pairwise comparison (according to the Wilcoxon criterion). The addition of Trp led to a significant increase of anxiety in terms of CA/OA ratio only in tetrahybrid mice but not in DBA/2J mice in the pairwise comparison between the 1st and the 2nd test.

### 2.4. Liver Histology

**Figure 6:**
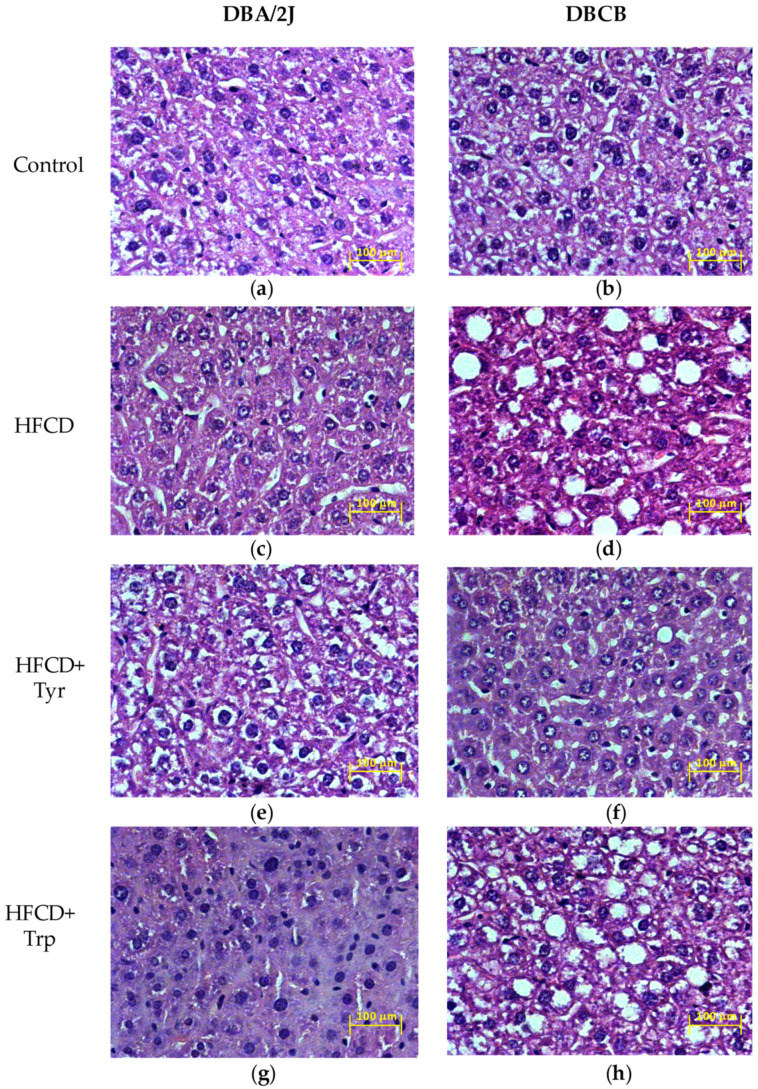
Representative light-optical micrographs of liver sections of mice fed: (**a**,**b**) Control diet; (**c**,**d**) HFCD; (**e**,**f**) HFCD with Tyr; (**g**,**h**) HFCD with Trp. DBA/2J mice (**a**,**c**,**e**,**g**); DBCB tetrahybrid mice (**b**,**d**,**f**,**h**). Stained with hematoxylin-eosin, magnification ×400.

One of the consequences of the consumption of HFCD in rodents may be the development of fatty degeneration of the liver. Microscopic examination of the liver (Figure 6) revealed a generally orthodox structure of the tissue in DBA2/J mice fed both the control diet and HFCD. The addition of Tyr increased fat accumulation in the perinuclear compartment of hepatocytes in these animals. In contrast, Trp consumption corresponded to the almost complete disappearance of fatty inclusions in the liver and the development of tissue edema with the closure of most of the lumen of the bile ducts. Moreover, the addition of Trp has contributed to the appearance of a large number of degrading micronucleus cells. In the tetrahybrids, signs of increased fat accumulation in the liver were observed even in the control group. At the same time, HFCD in tetrahybrids led to massive fatty degeneration of hepatocytes with the formation of rounded fatty vacuoles lacking internal structure. The addition of Tyr to HFCD seemed to weaken these changes, and the addition of Trp, on the contrary, aggravated them: along with the vacuoles, many cells appeared with deposition of fat in the perinuclear district.

### 2.5. Blood Biochemical Indices

The development of diet-induced obesity is often accompanied with signs of dyslipidemia, changes in nitrogen exchange, and protein catabolism. Analysis of the biochemical blood plasma parameters showed that HFCD consumption led to an increase in glucose level (Table 1) in DBA/2J mice. Tetrahybrid mice were characterized by an initially elevated glycemia already in the control group (*p* < 0.001, ANOVA, by the genotype factor). The addition of amino acids did not cause further changes in this indicator. According to the factor analysis, the addition of Tyr and, to a lesser extent, Trp to the diets led to a significant increase in indexes of the protein metabolism, such as levels of total protein, albumin, and urea (*p* < 0.05, ANOVA). In DBA/2J mice, these effects were also manifested in paired comparisons of groups. The consumption of Tyr and Trp was accompanied by an increase in the concentration of total calcium and in DBA/2J mice, also phosphorus (in the case of Trp). Under the action of Tyr, an increase in alanine aminotransferase (AlAT) activity was noted in DBA/2J mice, whereas in tetrahybrids, it was, conversely, decreased in comparison with the group that consumed HFCD without additives. Any effects of Trp on this indicator were not observed. Aspartate aminotransferase (AsAT) activity was increased under the action of HFCD only in DBCB mice (ANOVA, *p* < 0.05 by the genotype*diet factor in the tyrosine experiment). As shown by the calculation of the AsAT/AlAT activity ratio (De Ritis ratio), this indicator was significantly reduced in mice of both strains fed with Tyr. Trp led to a significant decrease in the activity of creatine phosphokinase (CPK) in DBCB mice consuming HFCD and to a significant increase in the lipolytic activity in animals of both strains. In erythrocytes of mice treated with HFCD, there was a significant decrease in glutathione peroxidase (GPO) activity, and the addition of Trp led to the normalization of this indicator in the tetrahybrid mice.

### 2.6. Blood Cytokines and Adipokines Levels

**Figure 7:**
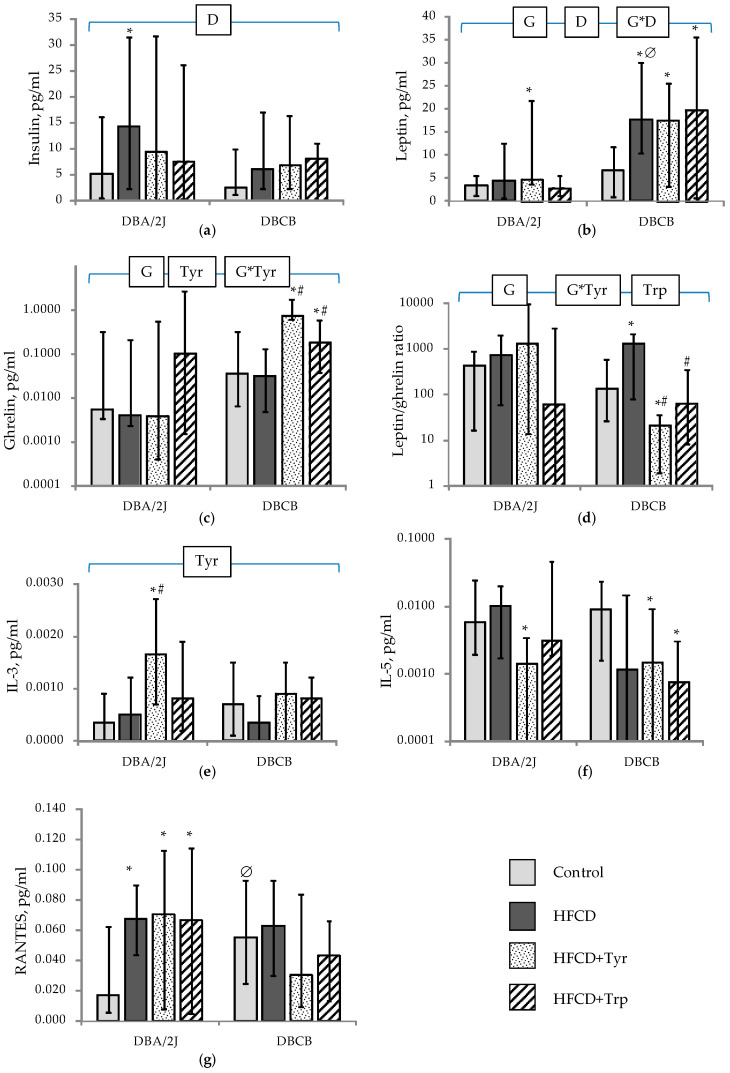
Levels of adipokines and cytokines (median; change intervals) in the blood plasma of mice when removed from the experiment on the 66th day: (**a**) Insulin; (**b**) Leptin; (**c**) Ghrelin; (**d**) Leptin/ghrelin ratio; (**e**) IL-3; (**f**) IL-5; (**g**) RANTES. * The difference with the control group is significant; # The difference with HFCD-only fed group is significant; ∅ The difference with DBA/2J mice is significant, *p* < 0.05, Mann–Whitney U-test. Horizontal bracket—distribution is non-uniform (*p* < 0.05, ANOVA) by the factors genotype (G), diet (D), tyrosine (Tyr), and tryptophan (Trp) and their combinations for the covered range of samples. The number of animals in groups—see Table 1.

The development of diet-induced obesity is a complex process involving local (in adipose tissue) and systemic inflammation, as evidenced by circulating levels of adipokines and cytokines. The consumption of HFCD led to a significant increase in mice insulin levels (Figure 7a); *p* < 0.05, ANOVA by diet (D) factor. At the same time, only in DBA/2J mice, this was combined with a significant increase in glucose levels (see Table 1), wherein DBCB mice were initially hyperglycemic, as was mentioned above (see Section 2.4). This could be a sign that glycemic control in tetrahybrid mice has been compromised due to their genetic background. No effect of the studied amino acids on the level of insulin and glycemia was found in mice of both genotypes.

The data on (Figure 7b) showed that all mice fed with HFCD showed a significant increase in the levels of circulating leptin (*p* < 0.05, ANOVA, by the diet factor), and the level of the latter was significantly higher in DBCB compared to DBA/2J mice (*p* < 0.05, ANOVA, by the genotype factor). The level of ghrelin (Figure 7c) increased in DBCB mice treated with Tyr and Trp supplements, and, consequently, the leptin/ghrelin ratio (Figure 7d) was the lowest in these animals. No significant effect of amino acids on the concentrations of IL-10, IL-12P (70), IL-17A, and MCP-1 was detected on the background of HFCD consumption (data not shown). At the same time, Tyr caused a significant increase in the IL-3 content (Figure 7e) and a decrease in IL-5 in DBA/2J mice (Figure 7f); in DBCB mice, both amino acids potentiated the effect of reducing the IL-5 level caused by HFCD. The level of RANTES (Figure 7g) was uniformly increased in all groups of DBA/2J mice receiving HFCD; in tetrahybrid mice, RANTES was significantly increased against DBA/2J when comparing control groups and did not significantly respond to the dietary interventions used.

## 3. Discussion

As a result of the research, differences were revealed in the effects of Tyr and Trp supplements in mice of two genotypes differing in the response to excess fat and carbohydrate intake (HFCD). DBCB tetrahybrid mice reacted to HFCD consumption differently than DBA/2J mice, which are one of their parent lines, due to the greater allelic diversity of the genome. In particular, HFCD possessing the increased energy density caused a decrease in palatability of food in tetrahybrid mice that led to the alignment of their specific energy consumption with the control group. At the same time, these mice had reduced mobility in the EPM test compared to DBA/2J and responded to the HFCD consumption by increasing fat deposition in the depot of white adipose tissue and hepatocytes (up to the development of balloon dystrophy). This also corresponded to the development of hyperleptinemia in these animals. A decrease in the relative proportion of brown fat in tetrahybrid mice indirectly indicates a possible inhibition of thermogenesis processes in them during the use of HFCD, and, therefore, a decrease in energy consumption during the consumption of a hypercaloric diet and, as a result, an increase in the relative weight of white fat. In contrast, DBA/2J mice that received HFCD were characterized by increased energy consumption but did not respond to a significant increase in bw and white fat, which may be a consequence of their increased energy spending. This is indicated by the greater relative proportion of thermogenic brown fat in total fat depots and increased activity in the EPM test. For DBA/2J mice, in contrast to DBCB, there was also a pronounced response of insulin and RANTES to the use of HFCD, which probably indicates a compensatory activation of fat catabolism processes in adipose tissue [16].

The study of the influence of tyrosine and tryptophan supplements on the vital indicators in mice was carried out taking into account the possible mechanisms mediated by biogenic amines—dopamine, catecholamines (in the case of tyrosine), and serotonin (in the case of tryptophan) both in the brain of animals and in peripheral organs. When assessing the central effects of amino acids, we used indicators of anxiety and search activity in the EPM test as well as muscle tone, measured in the grip force test. The latter indicator also may be indirectly related to the intensity of energy metabolism in muscle tissues, as is known from the literature [17]. Possible peripheral effects, allowing consideration of the influence of amino acids on the development of diet-induced obesity, were investigated using the assessment of integral, biochemical, and morphological parameters on the development of systemic inflammation accompanying the development of obesity using the analysis of adipokines and cytokines.

Taking into account the data received, the addition of Tyr did not have a significant effect in DBA/2J mice on feeding behavior (feed consumption) and accumulation of white and brown fat but contributed to an increase in muscle tone and potentiated anxiety enhancement in EPM. The reduction of the AsAT/AlAT ratio (De-Ritis [18]) under the influence of Tyr can be considered as a sign of a decrease in the intensity of protein catabolism when the transamination is reduced of aspartate with the formation of oxaloacetic acid (OAA) [19]. This is consistent with the increase in total protein levels in mice of both genotypes and albumin in DBA/2J mice receiving Tyr. An increase in urea levels in the same animals can be explained by increased deamination of an excess of aspartate in the urea cycle due to aspartate excess forming as a result of inhibition of its transamination into OAA.

The effect of Tyr on the liver was different in DBA/2J mice and tetrahybrids. If, in the first strain, the supplement led to an increase in AlAT activity and increased fat accumulation, in the second one, these changes had the opposite direction. Only in DBA/2J was a marked increase noticed in the level of IL-3 in combination with a decrease in IL-5. The discrepancy of these immune parameters, according to the literature, can occur at the level of a change in the ratio of specific receptors for IL-3 and IL-5 on eosinophils, which can be one of the signs of eosinophilic liver inflammation [20,21].

In DBCB mice, Tyr supplementation significantly prevented the development of fatty degeneration of hepatocytes caused by HFCD and contributed to the normalization of AlAT levels. This corresponded to the minimum value of the leptin/ghrelin ratio, which may indicate a weakening of the processes of lipogenesis [22]. Besides, Tyr exerted an influence on the state of the heart muscle in these animals, contributing to the normalization of organ mass and a decrease in CPK activity in the circulation.

The Trp supplementation did not affect the energy consumption of animals of both lines but increased muscle-contraction force and activated the processes of mineral metabolism, causing an increase in the levels of calcium and phosphorus in the blood plasma. The effect of Trp on the liver was ambiguous. In DBA/2J mice, it manifested, on the one hand, in the reduction of fat deposits and, on the other hand, in the development of edema and the appearance of micronucleus cells. In DBCB mice, Trp caused increased fat accumulation in the liver.

Thus, the features of the effect of Tyr supplementation on mice of the two lines used had both similarities and differences. In DBA/2J and DBCB mice, this supplement caused an increase in muscle tone, an increase in protein anabolic processes, and potentiated the effect of anxiety increase with the age of animals. Besides, in DBA/2J mice resistant to the development of diet-induced obesity, Tyr increased the toxic effect and fat accumulation in the liver, whereas, in spontaneously hyperglycemic and obesity-prone DBCB mice, these effects were opposite. These data are consistent with our previous results of a comparative assessment of integral, biochemical, and physiological parameters in DAT-KO knockout rats with genetic dopamine reuptake disorder and wild-type animals [23] and indicate, apparently, the ability of dietary Tyr to modulate dopamine exchange in the central nervous system.

Peculiarities of the action of the Trp supplement common to mice of both genotypes consisted in a muscle tone increase and potentiation of anxiety increase with the age of animals. The latter is in contradiction with a sedative effect postulated for Trp as a precursor of serotonin [11]. Trp influenced the mobility in the EPM test only in DBCB but not in DBA/2J mice. The differences between the two lines were manifested in the greater effect of Trp on the protein metabolism in DBA/2J mice and the increased accumulation of fat in the liver against the background of an increase in lipolytic activity in DBCB mice. Only in the latter, Trp showed signs of an antioxidant effect in terms of GPO activity and a partial cardioprotective effect (decrease in mortality, decrease in CPK activity, normalization of heart mass). The reasons for the Trp effects on behavioral reactions, metabolic processes, and toxic liver damage, which are complex and unequal for different mouse strains, should be sought apparently in the interaction of this amino acid with the intestinal microbiome of the animals. As it is known, indole and indolyl-3-propionic acid are among the main microbial metabolites of Trp [24]. The first one, entering the liver, is transformed there by the action of microsomal monooxygenases and sulfates into indoxyl sulfate, which has a toxic and prooxidant action [25]. The second metabolite, on the contrary, is considered as a trap of free radicals and can have a different, organ-protective effect under oxidative stress [26]. The ratio in the activity of different microbiota populations, alternatively synthesizing these metabolites, can vary significantly in mice of different strains [27], which partially explains the identified interlinear differences.

## 4. Materials and Methods

### 4.1. Animals and Experimental Design

The amino acids Tyr and Trp were purchased from Wirud Co (Bad Homburg, Germany), with a purity of 99.6% according to the manufacturer. The degree of amino acid purity was additionally checked by HPLC according to [28].

Male mice of inbred line DBA/2J, which were obtained from the nursery Stolbovaya (Stolbovaya, Moscow region, Russia), and a complex hybrid of the 2nd generation (referred to here as tetrahybrid) DBCB bred independently by crossing 4 different inbred lines of mice (DBA/2J, BALB/c, CBA/lac, and C57Black/6J) were used. The age of the animals at the beginning of the experiment was 8–9 weeks; the initial body weight (bw) was 24 ± 2 g. The method for breeding hybrid mice was presented earlier [15]. The work with animals was carried out following the rules of good laboratory practice [29] and in accordance with the Order of the Ministry of Health of the Russian Federation No. 199n of 1 April 2016, “On the approval of rules of good laboratory practice”. The design of the experiment was approved by the Ethics Committee of the Federal Research Centre of Nutrition and Biotechnology (protocol No. 4 of 20 April 2017).

DBA/2J mice and DBCB tetrahybrid mice were divided into 4 groups of 8 individuals not differing in the mean initial bw within each genotype (*p* > 0.05; ANOVA). Mice of the 1st (control) groups received a balanced, semi-synthetic diet for rodents corresponding to AIN93M [30], with a content of 10% fat by weight, and drinking water purified by reverse osmosis; the 2nd groups received a high-carbohydrate, high-fat diet (HFCD). Part of the starch in HFCD was replaced with fat (a mixture of 1:1 refined vegetable oil and pork lard) to a total fat content equal to 30% by weight of the dry substances of the diet, and drinking water was replaced with a 20% aqueous solution fructose. The 3rd and 4th groups received the same HFCD diet with the addition of Tyr and Trp in the estimated doses of 1250 and 250 mg/kg of bw, respectively. The indicated doses corresponded to a 2-fold increase of both amino acids that were received from the dietary casein and apparently had no large influence on diet palatability, meaning the bitter taste of these amino acids. Mice were kept four individuals to a cage at a temperature of 21 ± 1 °C and a light/dark mode of 12/12 h. The animals were fed diets for 65 days; moreover, food and liquid consumption were determined daily, bw was measured weekly, and the appearance, activity, and behavior were monitored.

### 4.2. Assessment of Behavioral Responses, Anxiety, and Muscle Tone Indices

Behavioral reactions (locomotor activity and anxiety levels) were studied in the elevated plus maze (EPM) installation (Panlab Harvard Apparatus company, Barcelona, Spain) on the 8th and 59th days of the experiment by the techniques described earlier [15].

### 4.3. Assessment of Integral and Biochemical Indices

Animals were removed from the experiment on the 66th day by exsanguination from the inferior vena cava under ether anesthesia. Blood was collected in measuring tubes with 0.3 mL of 1% heparin, fixing the dilution of each sample. The liver, spleen, heart, thymus, interscapular brown, and retroperitoneal white fat were collected and weighed on a laboratory balance with an accuracy of ±0.01 g. Liver tissue samples about 5 mm in diameter were fixed in a 3.7% formaldehyde solution in 0.1M sodium phosphate buffer pH 7.00 ± 0.05, dehydrated in alcohols of increasing concentration, impregnated with xylene, and filled in with a homogenized Histomix^®^ paraffin medium (BioVitrum, St. Petersburg, Russia). Paraffin sections 3–4 µm thick were made on a Microm HM355s microtome (Leica microsystems, Wetzlar, Germany), stained with hematoxylin and eosin using a standard technique, and examined in an AxioImager Zl microscope (Zeiss, Oberkochen, Germany) with a digital camera at magnification ×400. Plasma biochemical parameters were determined on a Konelab 20i biochemical analyzer (Thermo Fisher Scientific, Waltham, MA, USA). Standard methods such as kinetic methods recommended by the International Federation of Clinical Chemistry and Laboratory Medicine for alanine aminotransferase (AlAT), aspartate aminotransferase (AsAT), creatine phosphokinase (CPK), and total lipase activity were used. Glucoseoxidase enzymatic method for glucose, Trinder reaction with glycerol-3-phosphate oxidase for triglycerides, UV kinetic urease method for urea, Biuret test for proteins, a potentiometric method with ion-selective electrode for calcium, and spectrophotometric method for phosphorus were used also. The erythrocyte glutathione peroxidase (GPO) activity was determined by a direct spectrophotometric endpoint method using reduced glutathione as a substrate with Ellmann’s reagent in the presence of sodium azide. 

### 4.4. Cytokines and Adipokines Analysis

Determination of cytokines IL-3, IL-5, IL-10, IL-12p70, IL-17A, MCP-1, and RANTES and peptide hormones—ghrelin, insulin, and leptin—in the plasma of mice was performed by multiplex immunoassay with the basic set of Bio Bio-Plex Pro™ Reagent Kit V supplemented with the following reagents: Bio-Plex Pro™ Mouse Cytokine IL-3 Set, Bio-Plex Pro™ Mouse Cytokine IL-10 Set, Bio-Plex Pro™ Mouse Cytokine IL-12p70 Set, Bio-Plex Pro™ Mouse Cytokine RANTES Set, Bio-Plex Pro™ Mouse Diabetes Set, Bio-Plex Pro™ Mouse Diabetes Insulin Set, and Bio-Plex Pro™ Mouse Diabetes Leptin Set. All are manufactured by Bio-Rad Laboratories, Inc. (Hercules, CA, USA). Studies were run on a Luminex 200 multiplex analyzer (Luminex Corporation, Austin, TX, USA) using xMAP technology using Luminex xPONENT Version 3.1 software.

### 4.5. Statistical Analysis

Data were processed statistically using the ANOVA multivariate analysis of variance based on dietary intake factors (standard diet/HFCD), animal genotype, and the presence of Tyr and Trp in the diet; Wilcoxon–Mann–Whitney nonparametric test was used for pairwise comparisons; Student’s *t*-test was used for pairwise related quantities for daily energy intake; the SPSS 20.0 (IBM Corp., Armonk, NY, USA): software package was used. Differences were considered significant when the probability of accepting the null hypothesis was less than 0.05.

## 5. Conclusions

Adding amino acids Tyr and Trp to a high-carbohydrate, high-fat diet has a modulating effect on behavioral reactions, signs characterizing the development of alimentary obesity and liver damage, nitrogen metabolism, and the ratio of leptin and ghrelin production, which is most pronounced in DBCB mice that are sensitive to the harmful effects of this diet. 

The largest differences in the response of mice of the two lines to the consumption of amino acid supplements related to the effects on the level of locomotor activity and anxiety, liver tissue morphology, and cytokine production. This indicates genotypically determined features of the exchange of aromatic amino acids in mice of two strains, which leads, apparently, to differences in the synthesis and degradation of regulatory biogenic amines, including dopamine and serotonin, as well as some other biologically active and potentially toxic metabolites. Similar genotypic differences that take place in the human population probably present a source of ambiguity in the results of amino acid, as well as other biologically active substances used as supplements in therapeutic and preventive nutrition. Thus, the data obtained indicate the need to apply several qualitatively different in vivo models of obesity and metabolic syndrome for preclinical evaluation of the efficiency of biologically active substances in personalized therapeutic nutrition in patients with disorders related to excess nutrition. 

## Figures and Tables

**Table 1:** Biochemical parameters of the blood plasma of mice fed experimental diets, M ± S.E.M *.

Genotype	DBA/2J				DBCB				Factor ***
Group No	1	2	3	4	5	6	7	8	
Diet	Control	HFCD	HFCD + Tyr	HRCD + Trp	Control	HFCD	HFCD + Tyr	HRCD + Trp	
Number of Animals	8	7	8	8	7	4	7	7	
Glucose, mmol/L	12.8 ± 0.7 ^2–8^*	16.2 ± 0.9 ^1^	17.8 ± 0.8 ^1^	19.8 ± 1.9 ^1^	20.6 ± 1.8 ^1^	22.1 ± 2.5 ^1^	20.2 ± 1.8 ^1^	20.2 ± 0.7 ^1^	G
Protein, g/L	52.0 ± 2.5 ^3,4^	52.9 ± 2.0 ^3,4^	61.1 ± 1.2 ^1,2^	59.8 ± 2.4 ^1,2^	58.0 ± 3.6	56.1 ± 4.3	61.5 ± 1.2	60.1 ± 1.0	Tyr
Albumin, g/L	25.7 ± 1.2 ^3,4^	25.0 ± 1.9 ^3^	30.1 ± 0.7 ^1,2^	29.6 ± 1.4 ^1^	31.0 ± 1.2	29.7 ± 2.0	32.1 ± 0.6	32.7 ± 0.5	Tyr, Trp
Urea, mmol/L	5.15 ± 0.45 ^3,4^	5.18 ± 0.34 ^3,4^	7.27 ± 0.43 ^1,2^	6.54 ± 0.32 ^1,2^	6.63 ± 0.42	5.57 ± 0.32 ^7,8^	7.03 ± 0.46 ^6^	6.59 ± 0.22 ^6^	G ****, Tyr, Trp
Calcium, mmol/L	1.86 ± 0.09 ^3,4^	1.86 ± 0.10 ^3,4^	2.35 ± 0.12 ^1,2^	2.34 ± 0.14 ^1,2^	2.15 ± 0.06	1.95 ± 0.19 ^8^	2.30 ± 0.08	2.26 ± 0.06 ^6^	Tyr, Trp
Phosphorus, mmol/L	2.51 ± 0.19 ^4^	2.95 ± 0.19	3.06 ± 0.26	3.72 ± 0.32 ^1^	3.30 ± 0.20	3.03 ± 0.36	3.14 ± 0.23	3.37 ± 0.19	Trp
AlAT, U/mL	22.0 ± 3.8 ^3^	27.5 ± 11.1	50.9 ± 13.4 ^1^	35.3 ± 8.6	19.6 ± 4.9 ^6^	62.8 ± 20.5 ^5^	26.9 ± 6.2	47.7 ± 16.2	D, G × Tyr
AsAT, U/mL	220 ± 43	201 ± 39	205 ± 29	258 ± 66	180 ± 35	454 ± 253	191 ± 39	220 ± 38	G × D
AsAT/AlAT	11.2 ± 2.0 ^3^	10.6 ± 2.5 ^3^	5.0 ± 0.8 ^1,2^	9.9 ± 2.6	12.5 ± 5.1	10.1 ± 4.6	5.1 ± 0.3	12.3 ± 4.7	Tyr
KPK kU/mL	5.7 ± 1.7	5.9 ± 1.6	5.9 ± 0.9	7.1 ± 1.9	5.1 ± 0.7	10.7 ± 3.7 ^7^	3.6 ± 1.0 ^6^	5.6 ± 1.4	Tyr
Lipase U/mL	129 ± 13	110 ± 4 ^4^	120 ± 3	138 ± 9 ^2^	119 ± 15	111 ± 7 ^8^	148 ± 20	140 ± 8 ^6^	Trp
GPO mmol/min/ mg protein **	0.71 ± 0.02 ^2,3,4^	0.39 ± 0.03 ^1^	0.40 ± 0.03 ^1^	0.46 ± 0.01 ^1^	0.68 ± 0.03 ^6,7^	0.44 ± 0.03 ^5,8^	0.49 ± 0.06 ^5^	0.60 ± 0.02 ^6^	G, D, Trp

* Superscripts are group numbers, the difference with which is pairwise significant, *p* < 0.05, non-parametric Mann–Whitney test. ** In erythrocytes, the number of animals in groups of 6. *** Factors significantly influencing the distribution of indicator presented between groups of animals, *p* < 0.05, ANOVA, such as genotype of the animals (G), diet type (D), and amino acid added (Tyr, Trp). **** Only in experiment with Trp. See all abbreviations in Materials and Methods section.

## Data Availability

The datasets generated during the current study are available from the corresponding author on reasonable request by email.
